# Seasonal variation in the morphokinetics of *in-vitro*-derived bovine embryos is associated with the blastocyst developmental competence and gene expression

**DOI:** 10.3389/frph.2022.1030949

**Published:** 2022-11-03

**Authors:** Shira Yaacobi-Artzi, Dorit Kalo, Zvi Roth

**Affiliations:** Department of Animal Sciences, Robert H. Smith Faculty of Agriculture, Food and Environment, the Hebrew University, Rehovot, Israel

**Keywords:** morphokinetic, time-lapse system, embryo development, blastocyst, gene expression, seasonal effect

## Abstract

Summer heat stress is a major cause of reduced development of preimplantation embryos. Nevertheless, seasonal effects on embryo morphokinetics have been less studied. We used a non-invasive time-lapse system that allows continuous monitoring of embryos to study the seasonal impact on embryo morphokinetics. The experiments were performed during the cold and the hot seasons. Cumulus-oocyte complexes were aspirated from ovaries, *in-vitro*-matured, and fertilized. Putative zygotes were cultured in an incubator equipped with a time-lapse system. The cleavage and blastocyst formation rates were lower in the hot vs. the cold season (*p* < 0.01). The kinetics of the embryos differed between seasons, reflected by a delay in the second cleavage in the hot vs. the cold season (*p* < 0.03). The distribution of the embryos into different morphological grades (good, fair, and poor) throughout the first three cleavages differed between seasons, with a higher proportion of good-grade embryos in the hot season (*p* < 0.03). Cleaved embryos were categorized as either normal or abnormal, based on their first cleavage pattern. Normal cleavage was defined as when the first cleavage resulted in two equal blastomeres and further classified as either synchronous or asynchronous, according to their subsequent cleavages. Abnormal cleavage was defined as when the embryo directly cleaved into more than two blastomeres, it cleaved unequally into two unevenly sized blastomeres, or when the fusion of already divided blastomeres occurred. The proportion of abnormally cleaved embryos was higher in the hot season vs. the cold one (*p* < 0.01), reflected by a higher proportion of unequally cleaved embryos (*p* < 0.02). In the cold season, abnormally cleaved embryos had a lower potential to develop into blastocysts relative to their normally cleaved counterparts (*p* < 0.001). Blastocysts that developed in the cold and the hot seasons differed in the expression of genes that related to the cell cycle (*STAT1*; *p* < 0.01), stress (*HSF1*; *p* < 0.03), and embryo development (*ZP3*; *p* < 0.05). A higher expression level was recorded for the *STAT1* and *UHRF1* genes in blastocysts that developed from unequally vs. the synchronously cleaved embryos (*p* < 0.04). We provide the first evidence for a seasonal effect on embryo morphokinetics, which might explain the reduced embryo development during the hot season.

## Introduction

Low fertility of dairy cattle during the summer is a well-documented phenomenon. The mechanism by which summer heat stress impairs embryonic development seems to be multifactorial in nature and includes alterations in the follicle and its enclosed oocytes (1). As a result, a lower proportion of oocytes is fertilized, cleaved, and further develop to blastocysts during the hot, relative to the cold season (2). Exposing bovine oocytes to physiological relevant heat shock disrupts the nuclear and cytoplasmic maturation of the oocyte (3, 4), impairs the oocyte's mitochondrial functioning, alters the transcriptional expression in the developing embryos (2), and increases the proportion of oocytes that undergo apoptosis (3). In a previous study we reported that exposing oocytes to heat shock (41.5°C) during maturation further impairs the cleavage timing of the developed embryo (5). Nevertheless, the effect of the season on embryo morphokinetics has never been completely characterized for bovine embryos.

In humans, assisted reproduction technology such as the time-lapse system (TLS), has been established and widely used to select the best embryo for transfer, based on morphokinetic parameters (6–9). The time-lapse system is a non-invasive automated tool that enables the continuous recording of embryonic development, thereby enabling one to accurately time the mitotic divisions of blastomeres and to characterize the morphology of the cleaved embryos and that of the developed blastocysts (9–13). Among the morphokinetic parameters, the time of the first cleavage has been found to be a good parameter to predict the embryo developmental potential. Early cleaved embryos are more likely to develop to the blastocyst stage than their counterparts, the late-cleaving embryos (14, 15). Recent studies in bovines reported the abnormal patterns of the first cleavage. These include direct cleavage, unequal cleavage, and reverse cleavage (16, 17), which were found to have a lower developmental competence (13, 16, 18) and a reduced potential to be implanted (13). Recently, we found that under normothermic conditions, *in-vitro*-derived embryos that differed in their morphokinetics also expressed a differential transcriptomic profile. For instance, directly cleaved embryos differ from synchronously and asynchronously cleaved embryos by their expression of the 895 and 643 genes, respectively. The differentially expressed genes were involved in various biological pathways, such as the cell cycle, cytoskeleton regulation, apoptosis, and metabolism (18). In another study, exposing oocytes to heat shock (41.5°C) during *in-vitro* maturation resulted in a 4 and 5.5 h delay of the first and the second divisions, respectively. The median time at which blastocysts were formed was 6 h longer for the heat shock, relative to the control group (5). In light of these findings, the aim of the current study was to examine the seasonal effects (hot vs. cold) on the embryo morphokinetics by using the time-lapse system. We hypothesized that heat-induced alteration in the ovarian pool of oocytes will carry over and further affect embryo morphokinetics and reduce embryonic development during the hot season.

## Materials and methods

### Materials

All reagents were purchased from Merck-Sigma (Rehovot, Israel) unless otherwise specified. All culture media were prepared in our laboratory, as was previously reported (2, 19). This included oocyte maturation medium, which consisted of TCM-199 with Earle's salts supplemented with 10% (v/v) heat-inactivated fetal bovine serum (Sartorius, Goettingen, Germany), 0.2 mM sodium pyruvate, 50 μg/μl gentamicin, 1.32 μg/ml porcine folltropin-V (Vetoquinol, Magny-Vernoi, France), and 2 μg/ml estradiol. HEPES–Tyrode's lactate (HEPES–TL), which was supplemented with 0.3% (w/v) bovine serum albumin (BSA), 0.2 mM sodium pyruvate, and 0.75 mg/ml gentamicin (HEPES-TALP). Sperm-TL (SP-TL), was supplemented with 0.6% BSA, 1 mM sodium pyruvate, and 0.2 mg/ml gentamicin (SP-TALP). *In-vitro* fertilization-TL (IVF-TL) was supplemented with 0.6% essential fatty acid-free BSA, 0.2 mM sodium pyruvate, 0.05 mg/ml gentamicin, 0.01 mg/ml heparin (IVF-TALP), and potassium simplex optimized medium (KSOM).

### Methods

#### *In-vitro* production of embryos

*In-vitro* production of embryos was performed as previously described (2, 19, 20). For all *in-vitro* embryo production processes, oocytes were aspirated from ovaries collected from slaughterhouse post-mortem cows only; thus, living animals were not used in the experiments. The *in-vitro* model of bovine embryo production was approved by the Ethics Committee of the Hebrew University of Jerusalem (AG-22-16883-1). Cumulus oocyte complexes (COCs) collected in the cold and hot seasons were examined morphologically and classified under the same criteria by the same person, as described previously (21). This examination included the number of layers of the cumulus surrounding the oocytes and their cytoplasmic homogenous granulation and ooplasma color. We used only grade-I COCs that are spherical, symmetrical, and intact oocytes with a uniform size, color, and texture of the ooplasm and that are entirely surrounded by at least three to five compact layers of cumulus cells. Grade-II COCs, i.e., partially denuded or grade-III COCs, i.e., expanded, denuded, and a partially degenerated complex, or grade IV COCs, i.e., with a totally degenerated complex were excluded from the experiments in both the cold and the hot seasons. Briefly, COCs were washed three times in HEPES –TALP; then groups of 30 COCs were transferred to 500 μl droplets of oocyte maturation medium and were incubated in humidified air with 5% CO_2_ for 22 h at 38.5° C. At the end of maturation, COCs were washed in HEPES–TALP and transferred in groups of 30 to four-well plates containing 600 μl of IVF–TALP per well and 25 μl PHE (0.5 mM penicillamine, 0.25 mM hypotaurine, and 25 μM epinephrine in 0.9% NaCl). Frozen semen collected from a single bull was obtained from “Sion” (Hafetz-Haim, Israel) and was used for the *in-vitro* fertilization. Spermatozoa were co-incubated with COCs for 18 h at 38.5°C in a humidified atmosphere of 5% CO_2_. After fertilization, putative zygotes were carefully pipetted to remove any remaining cumulus cells and adhering spermatozoa and cultured in KSOM as a pool of 10 embryos per well, for ∼190 h at 38.5°C, with 5% CO_2%_ and 5% O_2_ in a conventional incubator, or individually placed in a specially designed dish for the Miri® Time-Lapse Incubator (CultureCoin®; Esco Medical Group, Kringelled, Denmark). The CultureCoin was covered with mineral oil and was inserted into Miri® Time Lapse (TL; Esco Medical Group). Culturing within the conventional incubator, parallel to the Miri® TL, was conducted in order to get a higher number of blastocysts per season. Note that in a set-up study in which embryos were *in-vitro*-produced in a conventional incubator and compared with those cultured in an incubator equipped with a time-lapse system, the embryo developmental rate did not differ between the two systems (data are not shown).

#### Morphokinetic evaluation

In the time-lapse incubator, each individual embryo was monitored automatically every 5 min throughout the culture period (∼190 h). The images were taken through seven focal planes by a built-in Zeiss objective (×20) with a numerical aperture of 0.35 specialized for 635 nm illumination using red light. The individual time-lapse images were then assembled into AVI movies using Miri®TL software. All videos were assessed daily by the same person. Note that the morphokinetic analysis was conducted only on embryos that had a clear morphokinetic visualization through their development.

*Embryonic development*: The cleavage rate was calculated as the number of cleaved embryos that were recorded at 44 h post-fertilization (including 2- to 4-cell stage embryos) out of the total oocytes (19). The blastocyst formation rate was calculated as the number of embryos that developed to the blastocyst stage at 170–190 h post-fertilization out of the totally cleaved embryos.

*Kinetic records*: The embryonic developmental kinetics were monitored for each developmental stage. This included the intervals from fertilization (time 0) to the first, second, and third divisions, while taking into account the number of cells in each embryo, i.e., 2, 3, 4, 5, 6, 7, 8, 10, 12, and 16 cells. The time at which the blastocyst started to expand was defined as the time of the blastocyst formation.

*Cleavage pattern characterization*: The embryo was defined as either normally or abnormally cleaved, based on its first-division characteristics. An embryo was defined as normally cleaved if the first cell division resulted in two blastomeres of the same size. Normally cleaved embryos were further classified into sub-groups: synchronous or asynchronous cleaved embryos (Supplements 1, 2, respectively). Synchronously cleaved embryos were characterized by synchronous divisions into 4, 8, and 16 blastomeres; asynchronously cleaved embryos were characterized by at least one asynchronous cleavage resulting in embryos with 3, 5, 6, 7, 10, or 12 blastomeres. The abnormally cleaved embryos were classified as directly-, unequal-, or reverse-cleaved embryos. Abnormally cleaved embryos included (i) directly cleaved embryos, i.e., cleavage from one blastomere directly into three or more blastomeres, (ii) unequally cleaved embryos, i.e., cell cleavage into two unevenly sized blastomeres, and (iii) reverse cleaved embryos, i.e., reduction of number of blastomeres from two to one following the first division (Supplements 3, 4, and 5, respectively).

*Morphology characterization*: Cleaved embryos at three developmental stages (the 2-, 4-, and 8-cell stage) were morphologically classified and scored as good, fair, or poor according to the International Embryo Technology Society (IETS) classification guidelines (22): good embryos had a symmetrical and spherical mass, blastomeres with uniform size, color, and density, a smooth zona pellucida, and no concave or flat surfaces; fair embryos had moderately irregular blastomeres in shape, size, or color (at least 50% intact); poor embryos had blastomeres with major irregularities in shape or mass (at least 25% intact), size, or color.

The blastocyst morphology was classified based on the pattern and the organization of the inner cell mass (ICM) and the trophoblast (TE) cells. The blastocyst was defined as having good morphology when the ICM had many tightly packed cells and the TE cells had a cohesive epithelium; the blastocyst was defined as having fair morphology when the ICM exhibited a loose group of cells and the TE cells had a loose epithelium; the blastocyst was defined as having as poor morphology when the ICM exhibited very few cells and the TE was composed of a few large cells.

#### Gene expression analysis

Gene expression analysis was conducted first on samples of pooled blastocysts developed in a conventional incubator (3 blastocysts/sample; 10 samples per season), regardless of their morphokinetics. Another analysis was performed on single blastocysts that developed in the Miri®TL incubator and had an entire time-lapse developmental record. Blastocysts (*n* = 24) were categorized according to their first cleavage, i.e., normally or directly and unequally cleaved embryos. The normally cleaved embryos were categorized according to their subsequent cleavages, i.e., synchronously or asynchronously cleaved embryos.

Samples were washed in DNase-free PBS-PVP, snapped-frozen in liquid nitrogen, and stored at −80°C until RNA extraction. Poly(A) RNA was isolated using the Dynabeads mRNA DIRECT Kit according to the manufacturer's instructions (Life Technologies, Carlsbad, CA, USA) as previously described (23, 24). Briefly, samples were lysed and mixed with prewashed Oligo (dT)_25_ Dynabeads. Following mRNA binding, each sample was washed twice with buffer A, twice with buffer B, and finally, mRNA was eluted with 10 mM Tris–HCl. The purified mRNA was used as the template in cDNA synthesis with SuperScript® III Reverse Transcriptase (Life Technologies). RT-qPCR was carried out with primers for 6 genes; this included mitochondrially encoded NADH dehydrogenase 2 (*mtND2*; mitochondrial function), zona pellucida 3 (*ZP3*; embryo development), Heat shock protein 85 (*HSP85*; stress), Signal transducer and activator of transcription 1 (*STAT1*; cell cycle), Heat shock transcription actor 1 (*HSF1*; stress), Ubiquitin–like, with PHD, and ring finger domains 1 (*UHRF1*; cell cycle) (Table 1). The genes were selected based on our previous microarray analysis (18). The RT-qPCR was normalized using the internal reference gene *YWHAZ*. This gene was previously assessed and found to have a stable expression in the blastocyst stage, independent of the experimental treatment (5, 19, 23, 24). The primers were derived from bovine sequences found in Genbank and specific primer pairs were designed using Primer 3.0 software.

**Table 1:** Primers used for qPCR analysis.

Gene	Gene Bank Accession number	Primer	Sequence (5′→3′)	Amplicon Size (bp)
*STAT1*	NM_001077900	Forward	CACGATGGTCTCAGCTTTCA	212
* *		Reverse	TTCAAGGATGCTTTCGATCC	
*UHRF1*	NM_001103098	Forward	ACAGAGCTGGGCCTCTACAA	204
* *		Reverse	ATCGGTACTTAGGGGTCTTG	
*HSP85*	NM_140559	Forward	TAGCCATGAATCCCCAGAAC	103
* *		Reverse	TCGTGGTCACATGGATGAGT	
*HSF1*	NM_001076809	Forward	ATGAAGCACGAGAACGAGGC	112
* *		Reverse	GCACCAGCGAGATGAGGAACT	
*Mt-ND2*	NM_ 174,565	Forward	CATGCTCCGAAACTCTGACA	129
* *		Reverse	GCATTTACACAGGCCCCTAA	
*ZP3*	NM_173974	Forward	GCCTGTTCCTTCAGCAAGTC	79
* *		Reverse	CCTTGCTACAGCATCGACAG	
*YWHAZ*	NM_00174814	Forward	GCATCCCACAGACTATTTCC	124
* *		Reverse	GCAAAGACAATGACAGACCA	

RT-qPCR was conducted using the LightCycler® 96 system (Roche, Basel, Switzerland) with the SYBR® Green qPCRBIO SyGreen Blue Mix Hi-ROX Kit (PCR Biosystems, Ltd., London, UK) in a final volume of 20 μl containing ultrapure water (Biological Industries), 400 nM of each primer and 3 μl diluted cDNA (1:4, v/v). A negative control, without reverse transcriptase, was included to ensure the absence of DNA template contamination. The reaction efficiency ranged between 90% and 110% with R2 > 0.995. The amplification program included preincubation at 95°C for 10 s to activate taq polymerase, followed by 40 amplification cycles of denaturation at 95°C for 10 s and annealing–elongation at 60°C for 15 s. All samples were run in duplicate. The RT-qPCR data were analyzed according to the 2^−ΔΔCT^ method; they expressed the fold change of each selected gene within experimental groups. Data were normalized against the cold season in the pooled blastocysts and against the synchronously cleaved embryo that developed in the cold season (expression was set to 1). The fold change for each gene was subjected to one-way ANOVA, followed by Student's *t*-test.

### Experimental design

**Figure 1:**
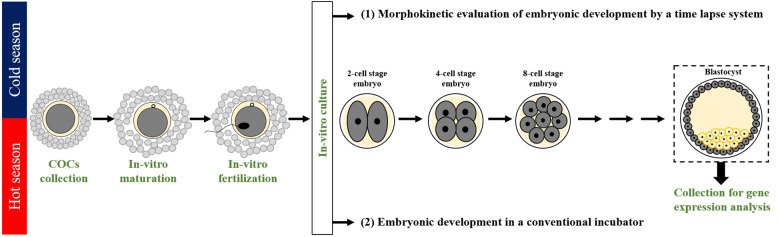
**Experimental design**. A seasonal study was performed in the cold and the hot seasons in the years 2018, 2019, and 2020. Cumulus-oocyte complexes (COCs) were aspirated from bovine ovaries collected at the slaughterhouse, *in-vitro* matured (22 h), and *in-vitro* fertilized (18 h). Then, putative zygotes were *in-vitro*-cultured: (1) individually for 190 h in an incubator equipped with a time-lapse system to allow non-invasive and continuous monitoring of the morphokinetic patterns throughout embryonic development. Morphokinetic monitoring included kinetic evaluation of the timing of the first, second, and third divisions and of blastocyst formation; morphological evaluation of embryos (good, fair, and poor) throughout the first, second and third divisions, as well as for the developed blastocysts; characterization of the first cleavage pattern into Normal or abnormal groups, followed by a further classification into subgroups: synchronous and asynchronous within the Normal, and direct, unequal, and reverse within the abnormal. Samples of individual blastocysts were subjected to mRNA extraction for gene expression analysis performed by RT-qPCR. (2) An additional experiment was performed in a conventional incubator throughout the cold and hot seasons. COCs were *in-vitro*-matured and fertilized; 10 putative zygotes were grouped and incubated in a conventional incubator for 190 h of culture. The developed blastocysts were pooled and subjected to mRNA extraction.

The experiments were performed for 3 consecutive years, 2018, 2019, and 2020, during the cold (Dec.–May) and hot (June–Sept.) seasons. A total of 1,676 COCs (677 COCs in the cold season and 999 in the hot season) were aspirated from ovaries collected from a slaughterhouse, *in-vitro*-matured (22 h), and fertilized (18 h); putative zygotes were individually cultured for 190 h in an incubator equipped with a time-lapse system for morphokinetic evaluation. This included the following: 1) kinetic monitoring of the first-, second-, and the third-cleaved embryos and blastocyst formation 190 h post-fertilization; 2) morphological evaluation of the cleaved embryos from the first-, second-, and the third-cleavages as well as for the developed blastocysts; and 3) characterization of the first cleavage pattern into either normal or abnormal with further classification into the subgroups of normal (i.e., synchronous or asynchronous) or abnormal (direct, unequal, or reverse) cleavages. The developed blastocysts were collected and subjected to mRNA extraction for gene expression analysis by RT-qPCR subjected to mRNA extraction for gene expression analysis by RT-qPCR (Figure 1). An additional experiment was performed in a conventional incubator throughout the cold and hot seasons. COCs were *in-vitro*-matured and fertilized; groups of 10 putative zygotes were incubated in a conventional incubator for 190 h of culture. For each season, samples of grouped blastocysts were subjected to mRNA extraction for gene expression analysis by RT-qPCR (Figure 1).

### Statistical analysis

The data included only embryos with a clear developmental record. In case of a partial or unclear record, embryos were excluded from the analysis. The proportion of embryos that cleaved at 44 h post-fertilization and that of the formed blastocysts were arcsine transformed and analyzed by JMP Pro-15 software (SAS Institute, Inc., 2004, Cary, NC). For each *in-vitro* production run, embryo developmental competence was analyzed by one-way ANOVA, followed by the Tukey–Kramer test. For each subgroup of embryos (normal, abnormal; synchronous, asynchronous; and direct, unequal, and reverse cleaved embryos), the variables included the percentages of cleaved embryos out of the total cleaved embryos and the proportion of blastocysts out of the cleaved embryos. Data are presented as the means ± SEM.

To compare the median time of the first-, second-, and third cleavages and the time of blastocyst formation, the Kruskal Wallis test, followed by the Wilcoxon test for pairwise comparisons, was used. Data for embryo kinetics are presented in box and whisker plots, indicating the timing for 25, 50 (i.e., median) and 75% of the cleaved embryos. Embryo morphology status (corresponding to good, fair, and poor) for cleaved embryos (first-, second-, and third cleavages) and for blastocysts was performed using Pearson's chi-square test.

For all analyses, *p* *<* 0.05 was considered significant. *p*-values of 0.05 and 0.1 were also reported as trends that might be realistic and worth noting.

## Results

### Environmental data

Environmental data for 2018–2020 were obtained from the central meteorological station in Bet-Dagan, Israel (Table 2). The average air temperature and relative maximum humidity were 18.1°C and 62.4% during the cold season and 26.5°C and 64.9% during the hot season, respectively. The calculated temperature humidity index was 75.2 and 62.5 units for the hot and cold seasons, respectively. Note that a THI value above 72 units indicates environmental thermal stress, since lactating cows undergo hyperthermia under those conditions (25).

**Table 2:** Environmental data obtained from the central meteorological station in Bet-dagan, Israel during the summer and winter (2018–2020).

Year	Season	Minimum average air temperature (°C)	Relative maximum humidity (%)	Thermal heat index (THI)
2018	Cold	20.6	59.5	65.7
	Hot	26.9	65.5	75.9
2019	Cold	16.0	64.0	59.7
	Hot	26.3	64.5	74.9
2020	Cold	17.7	63.8	62.3
	Hot	26.3	64.9	74.9

### Seasonal effect on embryo development

**Figure 2:**
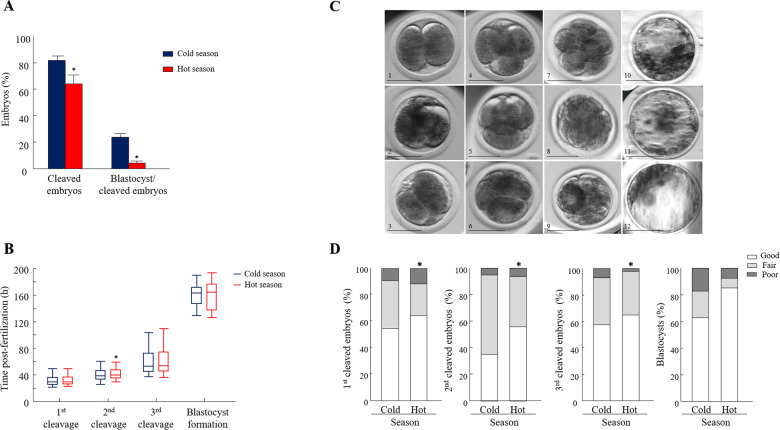
**Seasonal effect on oocyte developmental competence and morphokinetics**. (**A**) Cleavage and blastocyst formation rates calculated from the cleaved embryos (*n* = 1676, 677 COCs were collected in the cold season and 999 COCs were collected in the hot season). Data are presented as the means ± SEM. (**B**) Embryo kinetics was based on the timing post-fertilization of the first, second, and the third divisions and the time of blastocyst formation (*n* = 559 synchronously cleaved embryos). Data are presented in whisker plots and indicate the maximum and minimum values within the acceptable range defined by the two quartiles. Boxes indicate the 25th and 75th percentiles, and the middle-horizontal line indicates the median. The line inside the box is the median; the upper whisker is the 95th percentile values and the lower whisker is the 10th percentile. (**C**) Representative morphology images of (1) a 2-cell-stage embryo with equally sized blastomeres (good); (2) a 2-cell-stage embryo with equally sized blastomeres and up to 20% fragmentation or more (fair); (3) a 2-cell-stage embryo with unequally sized blastomeres and >50% fragmentation (poor); (4) a 4-cell-stage embryo with four equally sized blastomeres (good); (5) a 4-cell-stage embryo with equally sized blastomeres and up to 20% fragmentation or more (fair); (6) a 4-cell-stage embryo with unequally sized blastomeres and >50% fragmentation (poor); (7) a 8-cell-stage embryo with equally sized blastomeres (good); (8) a 8-cell-stage embryo with unequally sized blastomeres and minor (<20%) fragmentation (fair); (9) a 8-cell-stage embryo with unequally sized blastomeres and >50% fragmentation (poor); (10) blastocysts with evident inner cell mass (ICM), with many tightly packed cells and many trophoblast (TE) cells that form a cohesive epithelium (good); (11) blastocysts with a few TE cells that form a loose epithelium (fair); (12) blastocysts with ICM presenting loosely grouped cells and TE composed of a few large cells (poor). bar = 50 μm. (**D**): Distribution of 2-, 4-, and 8-cell-stage embryos and blastocysts into the different morphological categories (good, fair, and poor). A Chi-square test, followed by Fisher's exact test, was used for pair comparison within each cleavage between seasons. *n* = 559 synchronously cleaved embryos; 101 synchronously blastocysts. *p < 0.05.

The cleavage and blastocyst formation rates did not differ over the years, expressed by season ×  year interaction (*p* = 0.79; *p* = 0.1). Consequently, the data collected over the three years and for each season were combined and further analyzed. Oocyte developmental competence was lower during the hot, relative to the cold season, manifested by a lower proportion of cleaved embryos and a lower blastocyst formation rate (*p* = 0.01 and *p* = 0.001, respectively; Figure 2A).

### Seasonal effect on embryo kinetics

The analysis was performed only for synchronously cleaved embryos that developed in Miri® TL and that had an entire developmental record. The findings revealed that the kinetic pattern of the embryos was affected by the season in a stage-specific manner. In particular, a delay in the timing of the second cleavage was recorded in the hot season relative to the cold season. The median time of the second cleavage, i.e., when 50% of the embryos cleaved, was 39.5 vs. 38.0 h post-fertilization for the hot and cold season groups, respectively (*p* = 0.03; Figure 2B). The kinetics of the first and third cleavages and the time of blastocyst formation did not differ between seasons (*p* = 0.8; Figure 2B).

### Morphology of the cleaved embryos and blastocysts

Synchronously cleaved embryos that developed in Miri®TL and that had an entire developmental record were analyzed. Embryos were classified into three morphological grades (good, fair, and poor; Figure 2C) and evaluated through the first, second, and third divisions and at the blastocyst stage. In general, the distribution of the 2-, 4-, and 8-cell-stage embryos into the morphology grades differed between seasons (*p* = 0.03; Figure 2D). Surprisingly, in the first cleaved embryos, the proportion of good-grade embryos was higher in the hot season relative to the cold one (*p* = 0.001; Figure 2D). This was also true in the second and third cleavages, manifested by a higher proportion of good-grade embryos in the hot relative to the cold season (*p* = 0.001; Figure 2D). However, the distribution of blastocysts into the different morphological grades did not differ between seasons (*p* = 0.1; Figure 2D).

### Seasonal effect on the cleavage pattern

**Figure 3:**
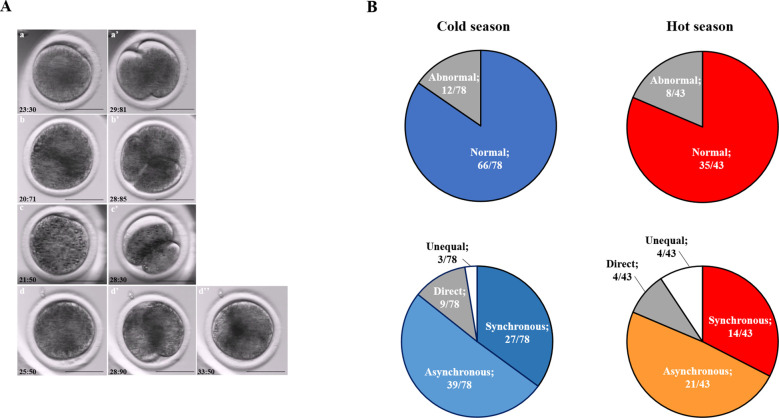
**Cleavage pattern of the first division.** (**A**) Representative images of the first cleavage pattern of normally and abnormally cleaved embryos. Presented are (a, a’) putative zygotes after fertilization that exhibited a Normal cleavage pattern and that are characterized by two equally sized blastomeres; putative zygotes that directly cleave, from 1 cell into 3 blastomeres (b, b’); putative zygotes that exhibited an unequal cleavage are characterized by two blastomeres of unequal size (c, c’); putative zygotes that exhibited a reverse-cleavage and are characterized by two blastomeres fused into one cell (d, d’, d'‘). Scale bar = 50 µm. (**B**) Pie charts demonstrating the distribution of the developed blastocysts into Normal and abnormal cleavages within each season (Upper panel). Further distribution of the developed blastocyst from normally (synchronous and asynchronous) and abnormally (direct, unequal) cleaved embryos within each season (Bottom panel). Presented are the total number of blastocysts in each group/subgroup.

Embryos that developed in Miri®TL and that had an entire developmental record were analyzed. Embryos from cold and hot seasons were defined as either normally or abnormally cleaved embryos, based on their first cleavage pattern. In addition, the normal and abnormal groups were further classified into subgroups. The normally cleaved embryos were classified as either synchronous or asynchronous subgroups. The abnormally cleaved embryos were classified as directly-, unequal-, or reverse-cleaved ones (Figure 3A).

The proportion of normally vs. abnormally cleaved embryos differed within and between seasons (*p* = 0.0001; Table 3A). This was manifested by a higher proportion of normally cleaved embryos. The proportion of embryos that further developed to the blastocyst stage was higher in the normally cleaved embryos relative to the abnormally cleaved embryos in the cold season (*p* = 0.009; Table 3A, Figure 3B), but it did not differ in the hot season (*p* = 0.3). Furthermore, the proportion of blastocysts that developed from normally cleaved embryos was higher in the cold season relative to their counterparts in the hot season (*p* = 0.0001; Table 3A, Figure 3B).

**Table 3:** Distribution of the cleaved embryos and blastocyst formation in normally and abnormally cleaving groups (3A) and subgroups (3B and 3C) during the cold and hot seasons.

**3A** **Season**	**Cold**		**Hot**		***P value***		
**Pattern of the first cleavage**	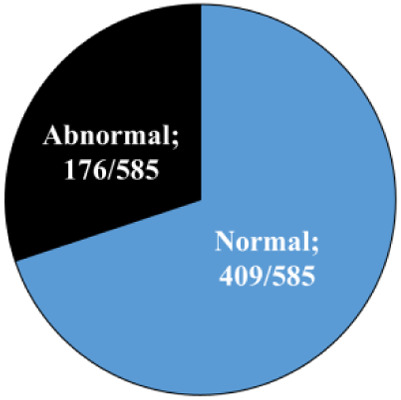		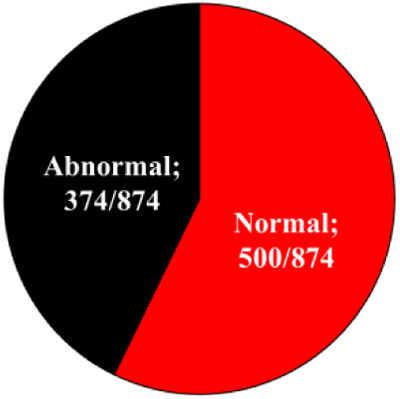				
**Subgroups**	**Normal**	**Abnormal**	**Normal**	**Abnormal**			
Cleaved embryo (%)	70.1 ± 2.8^a^	30.1 ± 2.9^b^	57.0 ± 2.5^c^	42.7 ± 2.5^d^	<0.0001		
Blastocysts/total cleaved (%)	12.5 ± 2.3^a^	1.5 ± 0.6^b^	3.7 ± 1.1^b^	0.7 ± 0.3^b^	<0.0001		
**3B** **Season**	**Cold**		**Hot**		***P value***		
**Pattern of the first cleavage**	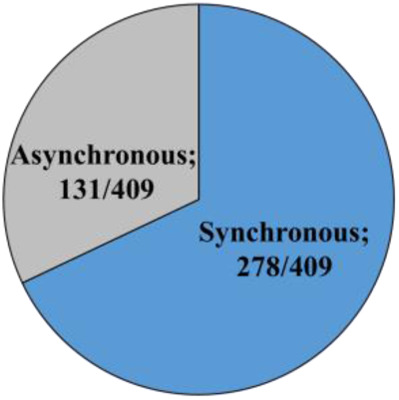		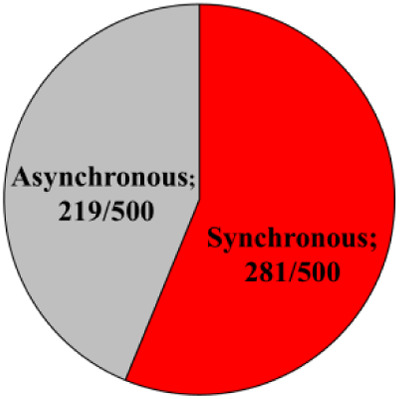				
**Subgroups**	**Synchronous**	**Asynchronous**	**Synchronous**	**Asynchronous**			
Cleaved embryos (%)	64.8 ± 5.5^a^	35.1 ± 5.5^c^	54.1 ± 5.0^ab^	44.7 ± 4.9^bc^	<0.0001		
Blastocysts/total cleaved (%)	8.8 ± 2.0^ab^	10.1 ± 2.6^a^	2.4 ± 0.8^b^	4.2 ± 1.3^ab^	<0.02		

**3C**
**3C Season**	**Cold**			**Hot**			***P value***
**Pattern of the first cleavage**	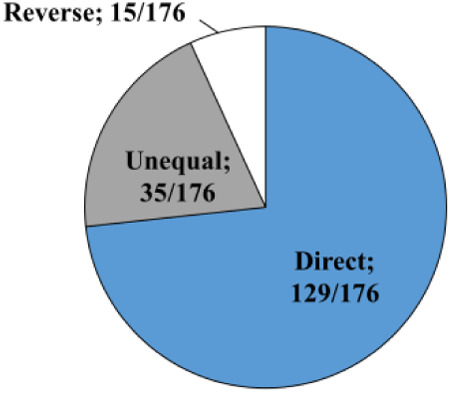			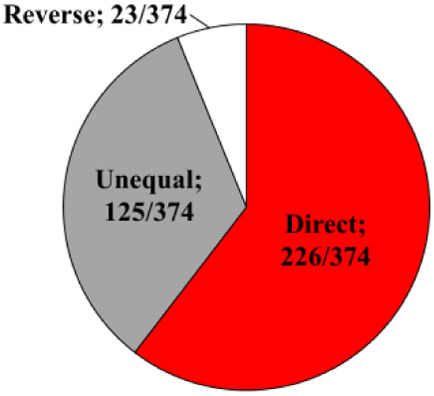			
**Subgroups**	**Direct**	**Unequal**	**Reverse**	**Direct**	**Unequal**	**Reverse**	
Cleaved embryos (%)	75.8 ± 3.8^a^	16.8 ± 3.6^d^	7.4 ± 2.4^d^	56.6 ± 5.1^b^	34.9 ± 4.8^c^	8.9 ± 4.9^d^	<0.001
Blastocysts/total cleaved (%)	4.2 ± 1.7^a^	1 ± 1^ab^	0 ± 0.0^b^	0.6 ± 0.4^ab^	0.7 ± 0.4^ab^	0 ± 0.0^b^	<0.01

Data were presented as the mean ± SEM calculated for each group/subgroup out of the total cleaved embryos within the group/subgroup, within and between the seasons.

^a-d^Different superscript letters indicate the statistical significance between groups within each row.

Among the normally cleaved embryos, a higher proportion of embryos were cleaved synchronously relative to the asynchronously ones in the cold season (*p* = 0.0001; Table 3B). However, no difference was found between the proportions of synchronously vs. asynchronously cleaved embryos in the hot season. With respect to blastocyst formation, no difference was found between the blastocysts that developed from synchronously or asynchronously cleaved embryos within seasons. However, the proportion of blastocysts that developed from asynchronously cleaved embryos in the cold season was higher relative to the synchronously cleaved embryos in the hot season (*p* = 0.02; Table 3B, Figure 3B).

Among the abnormally cleaved embryos, the distribution of cleaved embryos into direct, unequal, and reverse patterns differed between and within seasons (*p* = 0.0001; Table 3C). The proportion of directly cleaved embryos was higher within each season relative to the other abnormal subgroups (*p* = 0.01). In addition, the proportion of directly cleaved embryos was higher in the cold season relative to the hot season (*p* = 0.01). On the other hand, a higher proportion of unequally cleaved embryos was found in the hot season relative to the cold season (*p* < 0.02). The proportion of blastocysts that developed from each of the abnormal subgroups did not differ between seasons and was relatively low (*p* = 0. 1). Reverse-cleaved embryos did not develop to blastocysts in both the cold and the hot seasons (Table 3C, Figure 3B).

### Seasonal effect on gene expression in the developed blastocysts

**Figure 4:**
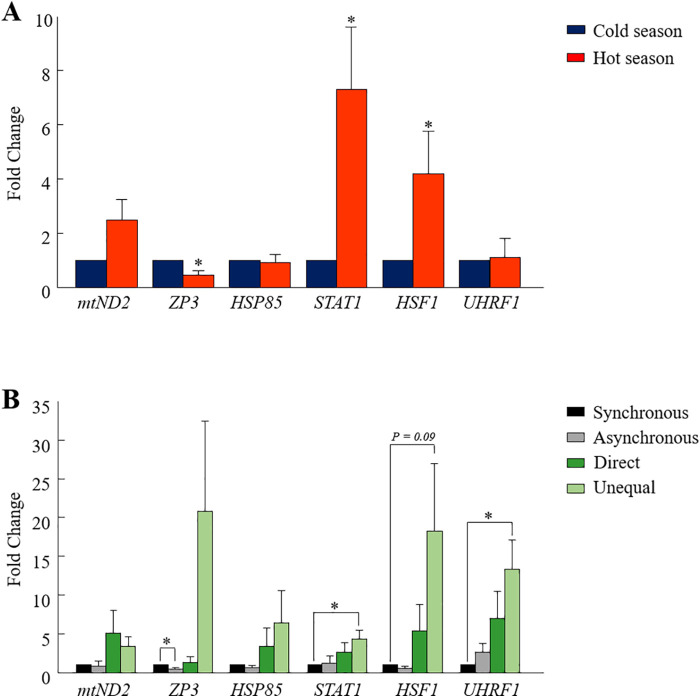
**Gene expression analysis in blastocysts.** (**A**) Cumulus-oocyte complexes were *in-vitro*- matured for 22 h and then fertilized. Putative zygotes were then *in-vitro*-cultured in a conventional incubator for an additional 190 h to allow blastocyst formation. The pools of blastocysts (*n* = 3 blastocysts per sample; 10 samples for each season) were collected and their mRNA was extracted for RT-qPCR, based on six selected genes (*mtND2, ZP3, HSP85, STAT1, HSF1,* and *UHRF1)*. Presented is the fold change calculated by the 2^−*ΔΔ*CT^ method, normalized against the reference genes *YWHAZ* and against the cold season (expression was set to 1). (**B**) Cumulus-oocyte complexes were *in-vitro*-matured for 22 h and then fertilized. Putative zygotes were *in-vitro*-cultured in a time lapse system for an additional 190 h to allow blastocyst formation. Blastocysts that developed from the synchronously-, asynchronously-, directly-, and unequally cleaved embryos were individually collected, and their mRNA was extracted for RT-qPCR. Gene expression was conducted based on six selected genes (*mtND2, ZP3, HSP85, STAT1, HSF1,* and *UHRF1).* Presented is the fold change calculated by the 2^−ΔΔCT^ method, normalized against the reference genes *YWHAZ* and against the synchronously cleaved embryo that developed in the cold season (expression set to 1). Data are presented as the means ± SEM. *p < 0.05.

One analysis was performed on pooled blastocysts that developed in a conventional incubator, regardless of their morphokinetics. The RT-qPCR findings revealed differential expression between seasons in some of the examined genes (Figure 4A). In particular, the expression of the *STAT1* and *HSF1* genes was higher in the hot season relative to that in the cold season (*p* = 0.01 and *p* = 0.05, respectively; Figure 4A). On the other hand, the expression of the *ZP3* gene was lower in the hot season, compared with the cold season (*p* = 0.01; Figure 4A). It is worth mentioning that the expression of the *mt-ND2* gene tended to be higher in the hot season relative to the cold season (*p* = 0.06, Figure 4A). However, the expression of the *HSP85* and *UHRF1* genes did not differ between the seasons (*p* = 0.8; Figure 4A).

### Association between cleavage pattern and gene expression in the developed blastocysts

Single blastocysts, developed in the TLS and classified according to their cleavage pattern (i.e., synchronous, asynchronous, direct, or unequal) were analyzed. The first analysis was performed while excluding the season, in order to explore differences in gene expression between cleavage patterns. The findings revealed the differential expression of some of the examined genes. In particular, a lower expression of the *ZP3* gene was found in blastocysts that developed from synchronously cleaved embryos relative to those that developed from asynchronously cleaved embryos (*p* = 0.02; Figure 4B). The expression of *mt-ND2, HSP85*, *HSF1, STAT1,* and *UHRF1* did not differ between the synchronously and asynchronously cleaved embryos (*p* = 0.2; Figure 4B). A prominent effect was recorded for the *UHRF1* and *HSF1* genes, with a higher expression in blastocysts that developed from unequally cleaved embryos relative to those that developed from synchronously cleaved embryos (*p* = 0.04 and *p* = 0.09, respectively; Figure 4B). The expression of *ZP3* and *STAT1* tended to be higher in blastocysts that developed from the unequally cleaved embryos relative to those that developed from synchronously cleaved embryos (*p* = 0.09, Figure 4B). The expression of *ZP3* and *HSP85* did not differ between the synchronously and unequally cleaved embryos (*p* = 0.1; Figure 4B). The expression of *mt-ND2, HSF1, ZP3, HSP85, UHRF1,* and *STAT1* did not differ between the synchronously and directly cleaved embryos (*p* = 0.2; Figure 4B).

A second analysis was performed to compare the gene expression within cleavage patterns and between seasons. In general, no differences were found (data not shown). Nevertheless, these findings should be taken with caution, because of the small number of blastocysts used in this analysis (*n* = 3 per subgroup). Note that collecting blastocysts from abnormally cleaved embryos is challenging, since their development is very low.

## Discussion

Season-induced disruption in the developmental competence of bovine oocytes seems to be multifactorial in nature. It includes direct or indirect damage to the oocyte and appears to involve more than one mechanism (3, 4, 19, 26, 27). Here we report on a seasonal effect on the morphokinetics of the developing embryo in association with a reduced formation of blastocysts and impaired gene expression in the formed blastocysts. The seasonal impact was manifested by a higher proportion of abnormally (mainly unequally) cleaved embryos, a delay in the second division, and by reduced blastocyst formation in the hot season relative to the cold season. A differential gene expression in the formed blastocysts was found between the seasons and within cleavage patterns. These impairments are suggested to be involved in the mechanism that underlies the reduced conception rates in dairy cows during the summer.

### Seasonal effect on kinetics, morphology, and developmental competence

A seasonal effect on oocyte developmental competence is a well-known phenomenon, manifested by a lower proportion of embryos that developed to the blastocyst stage during the hot season (19, 28, 29). In the current study, embryos were cultured in an incubator equipped with a time lapse system and the embryo analysis was individually performed. Similar to previous seasonal studies, reduced cleavage and blastocyst formation rates were recorded during the hot season, suggesting that the TLS experimental model used in the current study is correct. The findings revealed seasonal variations in the embryo kinetics, i.e., the timing of the first division, which was associated with the embryo's potential to develop to a blastocyst. Early stages of embryonic development are thought to be a key parameter for predicting embryos with a higher development potential. In particular, the timing of the first cleavage is thought to predict the human embryo quality, i.e., whether the embryo is transferable (30–32) or competent to survive cryopreservation (33). Previous studies in bovine, which were performed in an incubator equipped with a time-lapse system, found that the first three cleavages occurred 1 to 2 h earlier in embryos that later developed to the morula or blastocyst stages (34, 35). By using a time-lapse system, we recently found that under normothermic conditions, not only the timing of first cleavage, but also the timing of the second and the third cleavages is associated with the ability of the embryo to form blastocysts. The proportion of early cleaved embryos that developed to blastocysts was higher relative to the late cleaved embryos (18). An *in-vitro* study in which oocytes were exposed to heat shock (41.5°C) during maturation reported a 4- to 5.5-h delay in the timing of the first and the second cleavages, as well as the time of blastocyst formation (5). In the current study, we found a seasonal effect on the second cleavage, with a delay of 1.5 h, which was recorded in the hot, relative to the cold season. This was associated with a reduced potential to develop into the blastocyst stage. Taken together, the timing of the first divisions is considered a good predictor for the developmental competence of bovine embryos and was found to be affected by seasonal and environmental conditions.

The morphological grade of the embryo has been shown to correlate with its developmental competence and implantation potential (36, 37). In our previous study, performed under normothermic conditions, we found that embryos that expressed good morphology throughout the first three divisions further developed to blastocysts, whereas embryos with a lower-grade morphology did not (18). In light of the reduced cleavage rate found in the hot season, it is reasonable to assume that the developed embryo will have a lower morphological score. Surprisingly, this was not the case here, since cleaved embryos that developed from oocytes collected in the hot season presented a better morphological pattern, compared with those that developed in the cold season. Although not clear, it is suggested that oocytes that overcame the seasonal stress and underwent fertilization and further cleaved expressed good morphology during early embryonic development (the first, second and third divisions). However, the distribution of the blastocysts into the different morphological grades did not differ between seasons. On the other hand, our findings revealed a differential gene expression in blastocysts that developed in the cold season relative to the hot season. These findings suggest that while evaluating the quality of the embryo by morphology, the interpretation should be taken with caution since morphology has been previously reported to be misleading. Taken together, although blastocysts did not differ between seasons regarding their morphological pattern, they differed in their genetic profile, which might also be manifested in their implantation potential and further developmental stages.

### Seasonal effect on the transcript levels in the developing blastocysts

In a previous seasonal study, differential gene expression was reported for embryos that developed in the hot, relative to the cold season (19). For instance, a higher expression of the *GDF9* gene was found in the 4-cell stage and a lower expression of *POU5F1* in the 4-cell, 8-cell-stage embryos and blastocysts in the hot season (19). Accordingly, in the current study, we found a seasonal differential gene expression in blastocysts. One of the differentially expressed genes was the transcriptionally active heat shock transcription factor 1 (*HSF1*). Under stress conditions, heat shock factors (HSFs) are activated, enter the nucleus, and bind to the heat shock elements, whereas under normal conditions, *HSF1* stays in an inactive state in the cytoplasm (38, 39). Besides their roles in stress response, HSFs perform crucial roles during gametogenesis and development. The *HSF1* gene is essential for embryonic development and is involved in the apoptotic process and in establishing the chromatin structure and genome stability (40). *HSF1* plays a critical role in the regulation of oocyte meiosis; this can be linked to its transcriptional regulation of Hsp90*α*, a prominent chaperone expressed in oocytes (41). In mice, Hsf1-/-females do not produce viable blastocysts, since all embryos are arrested at earlier developmental stages, mostly at the 1–2-cell stage (42). Here we report that in the hot season the derived blastocysts exhibited a higher expression of the *HSF1* gene relative to those that developed in the cold season. Since the ovarian pool of oocytes was exposed to a seasonal stress, high expression of *HSF1* in the formed blastocysts might indicate a carryover effect from the oocyte to the embryo. Interestingly, a higher *HSF1* expression was found in blastocysts that developed from unequally cleaved embryos relative to those that developed from synchronously cleaved embryos. Thus, it is reasonable to assume that alterations in the *HSF1* expression underlie the unequal division. In support of this notion, *HSF1*-deficient oocytes exhibited a wide range of abnormal microtubular structures (43).

Differential gene expression between seasons was also recorded for the signal transducer and the activators of transcription 1 *(STAT1*), a gene that is associated with the cell cycle. *STAT1* is a member of the JAK/STAT signaling pathway; it plays an important role in fertility. Truchet et al. (44) reported that *STAT1* is expressed in mouse oocytes and in preimplantation embryos and that it has functional importance in early embryonic development due to its role in the cell cycle and apoptosis. The JAK/STAT pathway is constitutively activated in preimplantation mouse embryos, manifested by the phosphorylation of *STAT1, STAT2,* and *STAT3* (44). A study on Holstein cows found polymorphisms in *STAT1* and *STAT3* and that the interactions between the two genes significantly contribute to embryo survival (45). Here we found a higher transcript abundance of the *STAT1* gene in blastocysts that developed from unequally cleaved embryos relative to those that developed from synchronously cleaved ones. Higher *STAT1* expression has been associated with a different clinical outcome in cancer patients, indicating that patients with a high expression of *STAT1* in cancer tissues experience worse clinical outcomes, compared with patients with low expression levels (46, 47). Based on this, a high expression of *STAT1* is suggested to underlie this abnormal cleavage.

Another gene that is differentially expressed in blastocysts is the zona pellucida 3 gene (*ZP3*), manifested by a lower expression in the hot, relative to the cold season. *ZP3* is involved in the formation of the ZP3/ZP4 complex, which is responsible for the binding of the sperm to the oocyte (48). Previous studies in bovines suggested that the *ZP* subgroup of genes is downregulated following fertilization (48, 49). It is therefore possible that a low expression of *ZP3* in the oocyte underlies the reduced cleavage rate and embryonic development during the summer. In addition, in the current study, *ZP3* was lower in asynchronously relative to synchronously cleaved embryos. In a recent microarray analysis, we revealed that the expression of *ZP3* was higher in blastocysts that developed from directly relative to synchronously cleaved embryos (18). Taken together, it possible that high or low expression of *ZP3* affects to some extent the pattern of the first division and consequently embryonic development. To clarify this point, the expression of *ZP3* should be examined in the oocyte itself, rather than in the blastocyst, as performed in the current study.

Previous studies have shown that reduced developmental competence under elevated temperatures is strongly associated with seasonally induced alterations in mitochondrial features, such as mitochondrial distribution, the proportion of highly polarized mitochondria, and the expression of mitochondrion-associated genes (2); in particular, the expression of NADH dehydrogenase subunit 2 (*mt-ND2*) was higher during the summer relative to the winter. In the current study, the expression of *mt-ND2* tended to be higher in blastocysts that developed during the hot season, compared with the cold season. *mt-ND2* is part of complex I and is involved in proton translocation across the inner mitochondrial membrane. It is possible that exposing oocytes to thermal stress impairs the mitochondrial functioning, manifested by a higher expression of *mt-ND2.* In contrast, *in-vitro* exposure of bovine oocytes exposed to heat shock reduced the expression of *mt-ND2* in the blastocysts that developed (5). Although not clear, the differences between studies might be due to the experimental design, i.e., the seasonal vs. the *in-vitro* models.

### Association between cleavage patterns and blastocyst gene expression

With the increased use of the time lapse system in human IVF units, the pattern of the first divisions have received high attention and have become an accepted parameter to predict embryo development potential. Abnormal patterns such as direct-, reverse-, and unequal cleavages have been shown to correlate with lower embryo development and implantation potential (8). In addition, the occurrence of uneven cleavage through the preimplantation stages was found to have a negative impact on pregnancy outcome (50, 51). Similarly, in bovines, unequally cleaved embryos were associated with reduced blastocyst rates (35, 50). In our previous study, performed under normothemal conditions, unequal cleavage was found to be determinate, since only a few unequally cleaved embryos further developed to the blastocyst stage (18). Here we report, for the first time, that the pattern of the first divisions differs between seasons. The proportion of the normally cleaved, out of the total cleaved embryos was significantly higher than that of the abnormally cleaved embryos in both the hot and cold season. Of the abnormally cleaved embryos, the proportion of unequally cleaved embryos was higher in the cold relative to the hot season (14.3 vs. 5.9%, respectively).

The mechanism that underlies the abnormal division, in particular, the unequally cleaved embryos, is not clear. Blastomeres that were isolated from unequally cleaved human embryos had a higher number of blastomeres with numerical chromosomal aberrations, relative to the equally cleaved blastomeres (29.4 vs. 8.5%, respectively; (51). It was also reported that unequal blastomere size within an embryo is correlated with multinucleation (51), most likely due to the impaired migration of chromosomes through the mitotic anaphase (52, 53). It is suggested that with an unequal division the two resulting blastomeres receive unequal amounts of mRNA, proteins, or cell organelles such as mitochondria, which in turn, affect further embryo development. Although presently not clear enough, unequal division might involve alterations in pivotal genes that involve the cell cycle.

In a recent microarray analysis, we found a different transcriptomic profile between blastocysts that developed from normally vs. abnormally cleaved embryos (18). About 895 and 643 differentially expressed genes were found between blastocysts that developed from synchronously or asynchronously and directly cleaved embryos. The differentially expressed genes were related to the cell cycle, cell differentiation, metabolism, histone modification, and apoptosis. Similarly, in the current study, differential expression of genes related to the cell cycle, stress, and embryo development were found in blastocysts that developed from synchronously cleaved embryos relative to direct- or unequally cleaved embryos. In particular, a higher expression of the Ubiquitin-like, containing PHD and ring finger domains, 1 *(UHRF1*) gene was found in blastocysts that developed from unequally cleaved embryos relative to synchronously cleaved embryos. *UHRF1* is involved in multiple cellular functions such as proliferation, cell cycle regulation, and epigenetic modifications (54, 55). Alterations in the *UHRF1* levels can influence cell cycle progression, apoptosis, and response to DNA damage (54, 56, 57). Studies in tissue culture cells reported that *UHRF1* depletion causes cell cycle arrest (58, 59). In addition, oocytes derived from *UHRF1*-deficient mice exhibit increased oocyte aneuploidy, lead to disturbance of spindle and chromosome separation as well as DNA damage (60). In addition, disruption of the *UHRF1* function has a negative impact on the oocyte quality and the subsequent embryo development. In zebrafish, both high and low expression in *UHRF1* impair the rapid cell cycles and the massive epigenetic modifications that are required for normal progression through the maternal–zygotic transition (61). *In-vivo* and *in-vitro* studies in mice reported that *UHRF1*-deficient oocytes can be fertilized; however, embryos derived from these oocytes could not reach the blastocyst stage (60). Taken together, *UHRF1* is a pivotal gene that is involved in early embryonic development and it is suggested to be involved in the mechanism that underlies unequal division. This notion requires further investigation.

## Conclusions

Using the time-lapse system enabled us to explore the seasonal variations in oocyte developmental competence in association with morphokinetic alterations in the embryos that developed. In particular, the proportion of abnormally cleaved embryos was higher in the hot, relative to the cold seasons, mostly reflected by a higher proportion of unequally cleaved embryos. Seasonal alteration in the embryo morphokinetics at early developmental stages might explain, at least in part, the reduced pregnancy rate in dairy cows during the hot season.

## Data Availability

The raw data supporting the conclusions of this article will be made available by the authors, without undue reservation.

## Supplementary material

SUPPLEMENTARY VIDEO 5

SUPPLEMENTARY DATA SHEET 1
