# Supplementary material for: Seasonal variation in the morphokinetics of in-vitro-derived bovine embryos is associated with the blastocyst developmental competence and gene expression
Source: Front Reprod Health. 2022 Nov 3;4:1030949. doi: 10.3389/frph.2022.1030949 (PMC9670144; doi:10.3389/frph.2022.1030949)
Supplement: SUPPLEMENTARY DATA SHEET 1 [file Datasheet1.docx]

# Supplementary Movies

**Supplementary Movie 1.** Representative movie of a synchronously normal cleavage. Putative zygote that exhibited a synchronously cleavage pattern, characterized by first cleavage into two equally sized blastomeres that further cleaved into 2, 4, 8, and 16 blastomeres**.**

**Supplementary Movie 2.** Representative movie of an asynchronously normal cleavage. Putative zygote that exhibited an asynchronously cleavage pattern, characterized by first cleavage into 2 blastomeres followed by second cleavage of 1 blastomere resulting in embryos with 3 blastomeres.

**Supplementary Movie 3.** Representative movie of directly cleaved embryo. Putative zygote that directly cleave, from 1 cell into 3 blastomeres**.**

**Supplementary Movie 4.** Representative movie of an unequally cleaved embryo. Putative zygotes that exhibited an unequal cleavage and characterized by two blastomeres of unequal size.

**Supplementary Movie 5.** Representative movie of reverse-cleaved embryo. Putative zygote that exhibited a reverse-cleavage and characterized by a two blastomeres fusion into one cell.
